# Early and stable upregulation of collagen type II, collagen type I and YKL40 expression levels in cartilage during early experimental osteoarthritis occurs independent of joint location and histological grading

**DOI:** 10.1186/ar1471

**Published:** 2004-12-07

**Authors:** Helga Lorenz, Wolfram Wenz, Mate Ivancic, Eric Steck, Wiltrud Richter

**Affiliations:** 1 Division of Experimental Orthopedics, University of Heidelberg, Germany

**Keywords:** ACLT, cartilage, gene expression, histology, osteoarthritis

## Abstract

While morphologic and biochemical aspects of degenerative joint disease (osteoarthritis [OA]) have been elucidated by numerous studies, the molecular mechanisms underlying the progressive loss of articular cartilage during OA development remain largely unknown. The main focus of the present study was to gain more insight into molecular changes during the very early stages of mechanically induced cartilage degeneration and to relate molecular alterations to histological changes at distinct localizations of the joint. Studies on human articular cartilage are hampered by the difficulty of obtaining normal tissue and early-stage OA tissue, and they allow no progressive follow-up. An experimental OA model in dogs with a slow natural history of OA (Pond–Nuki model) was therefore chosen. Anterior cruciate ligament transection (ACLT) was performed on 24 skeletally mature dogs to induce joint instability resulting in OA. Samples were taken from different joint areas after 6, 12, 24 and 48 weeks, and gene expression levels of common cartilage molecules were quantified in relation to the histological grading (modified Mankin score) of adjacent tissue. Histological changes reflected early progressive degenerative OA. Soon after ACLT, chondrocytes responded to the altered mechanical conditions by significant and stable elevation of collagen type II, collagen type I and YKL40 expression, which persisted throughout the study. In contrast to the mild to moderate histological alterations, these molecular changes were not progressive and were independent of the joint localization (tibia, femur, lateral, medial) and the extent of matrix degeneration. MMP13 remained unaltered until 24 weeks, and aggrecan and tenascinC remained unaltered until 48 weeks after ACLT. These findings indicate that elevated collagen type II, collagen type I and YKL40 mRNA expression levels are early and sensitive measures of ACLT-induced joint instability independent of a certain grade of morphological cartilage degeneration. A second phase of molecular changes in OA may begin around 48 weeks after ACLT with altered expression of further genes, such as MMP13, aggrecan and tenascin. Molecular changes observed in the present study suggest that dog cartilage responds to degenerative conditions by regulating the same genes in a similar direction as that observed for chondrocytes in late human OA.

## Introduction

Osteoarthritis (OA) is a disease with a high prevalence, and it occupies a very important place in orthopedic surgery. It is characterized by progressive degeneration of articular cartilage and damage to subchondral bone. While macroscopic, histological and biochemical features of OA have been extensively studied [1-4], the molecular changes in chondrocyte metabolism underlying the pathophysiological process of cartilage degeneration remain largely unknown. Studies on human articular cartilage are hampered by the difficulty of obtaining normal tissue and early-stage OA tissue. For this reason a number of animal models have been developed in which cartilage degeneration is induced by causing permanent joint instability [5-7]. In the Pond–Nuki model in dogs, anterior cruciate ligament transection (ACLT) leads to joint laxity and altered mechanical loading in the knee joint, resulting in cartilage degeneration over time. This model has the advantage of a fairly slow natural history of OA, since full-thickness loss of articular cartilage does not develop until about 4–5 years after ACLT. The resulting degenerative changes in cartilage and synovial tissue thus closely resemble those in natural canine OA and human OA [1,4,6,8].

Recent studies have examined the gene expression levels of collagen type (col) II, aggrecan and small proteoglycans in dogs [9-11] and of several cartilage-related and OA-related molecules in rabbits [12-14] over time after ACLT. However, most of these studies used insensitive methods of RNA detection (northern blot) [9,11], or the methods were only semiquantitative [13,14] or failed to relate specific gene expression to the basic expression level of housekeeping genes [9,10]. This may be one reason why contradictory results have been obtained; for example in rabbits, in which no alteration of col II gene expression [12,13] or upregulation at region-specific sites [14] is reported. Other matrix molecules, such as aggrecan and fibromodulin, seem to be altered only at isolated time points [13,14]. Col II expression in dogs was reported to be higher in ACLT-treated knees than in control knees. These changes in col II expression progressed in one study [9] and decreased in another one [10], while the elevation of aggrecan was stable.

The histological appearance of OA cartilage makes it obvious that the severity of changes may vary with the location in the joint. It has been demonstrated in the rabbit knee joint that there are differences in RNA levels between different cartilage regions, which emphasizes that it may be risky to pool samples from distinct regions of the knee for molecular analysis [15]. Nonetheless, the difficulty of extracting sufficient RNA from a limited quantity of cartilage has hampered attempts to find direct correlations between the histological appearance of the cartilage and the metabolism of the chondrocytes in this region. For this reason, histological information has either not been considered at all [9,10] or has been derived from separate animals [12,14] in earlier gene expression studies, although region-specific histological progression of cartilage degeneration was reported in the rabbit ACLT model [14].

The aim of the present study was to perform a highly sensitive and quantitative molecular analysis of the response of articular cartilage to increased joint instability and altered mechanical loading, and thus to gain more insight into the very early stages of mechanically induced cartilage degeneration. The major objective was to focus on local aspects of gene expression with reference to the histological grading of cartilage degeneration. By improving RNA yields from small cartilage samples and selecting highly sensitive methods for quantitative gene expression analysis, we studied alterations in chondrocyte metabolism side by side with the histological appearance of cartilage degeneration in adjacent tissue. The Pond–Nuki model was chosen because of its close similarity to human OA, and the gene expression levels of six OA-related molecules were followed longitudinally over 48 weeks.

## Methods

### Animals

Twenty-four skeletally mature beagle dogs were each assigned randomly to one of four experimental groups. The animals' ages ranged from 1 to 2 years (average, 17 months) and the animal body mass was 15–22 kg (average, 19 kg). The dogs were cared for according to the guidelines of the local Council of Animal Care. ACLT was performed on each dog's left knee as described elsewhere [7], with the right knee serving as the control. After 3 days in individual indoor kennels the animals were allowed to move about freely in groups in outdoor pens. The dogs showed no abnormalities in posture and movement before surgery and at euthanasia. Dogs in the four groups were euthanized after 6, 12, 24 and 48 weeks, respectively.

### Preparation of specimens

Samples were taken within 2 hours after euthanasia. A full-thickness sample about 5 × 2 mm^2 ^in area, including cartilage and bone, was excised with a hammer and chisel from the lateral and the medial femoral condyle and from the lateral and the medial tibial plateau. For molecular analysis, articular cartilage was shaved off the articular surface 2–4 mm around the site of the histological samples and was immersed in liquid nitrogen. Peripheral areas of cartilage were not included.

### Histology

Samples were fixed in 4% formalin, embedded in paraffin, cut into 3-μm-thick slices and were stained with Safranin O. Specimens were analyzed for the degree of histological change using the Mankin score [16] modified as previously published [17]. All sections were graded by three independent observers blinded to the group, and median scores were determined for statistical analysis.

### Gene expression analysis

After measurement of the frozen tissue mass, cartilage samples were pulverized in a freezer mill (Dismembrator S; Braun Biotech, Melsungen, Germany). Messenger RNA was extracted from the powder using oligo(dT)-coated beads (Dynabeads; Dynal, Oslo, Norway) according to the manufacturer's instructions, and was quantified and tested for quality by measurement of the optical density at 280 and 260 nm in a NanoDrop ND100 photometer (Kisker, Steinfurt, Germany). First-strand cDNA was generated using reverse transcriptase (SuperScript II; Invitrogen Life Technologies, Karlsruhe, Germany) and oligo(dT) primers. The cDNA was further purified using a commercially available kit (PCR purification kit; Qiagen, Hilden, Germany). In a pilot study, tissue requirements had been minimized to confine the analysis to the close proximity around the histological tissue sample. About 50 mg tissue was sufficient for quantitative analysis of up to 10 genes.

Canine-specific PCR primers for GAPDH, col I, col II, aggrecan and MMP13 were designed on the basis of gene bank information. For tenascinC, YKL39 and YKL40 degenerated primers were applied on canine chondrocyte cDNA to obtain specific DNA fragments. Amplified fragments were purified and sequenced, and specific primers were designed based on this sequence: GAPDH, 5'-GATTGTCAGCAATGCCTCCT-3' and 5'-GTGGAAGCAGGGATGAT-GTT-3' ; col I A1, 5'-GAGAAAGAGGCTTCCCTGGT-3' and 5'-AGGAGAACCATCTCGTCCAG-3' ; col II A1, 5'-TGAATGGAAGAGCGGAGACT-3' and 5'-CCACCATTGAT-GGTTTCTCC-3' ; YKL40, 5'-TCTGTTGGAGGATGGAGCTT-3' and 5'-CAGCCTTCATTTCCTTGACC-3' ; MMP13, 5'-CAGAGCGCTACCTGAAATCC-3' and 5'-CATTGTACTCGCCCACATCA-3' ; aggrecan, 5'-ACCCCT-GAGGAACAGGAGTT-3' and 5'-GTGCCAGATCATCACCACAC-3'; tenascinC, 5'-AGGGGGTCTTCGACAGTTTT-3' and 5'-CATGGCTGTTGTTGCTATGG-3'.

Quantitative reverse transcriptase-PCR was performed in a LightCycler (Roche Diagnostics, Mannheim, Germany) with optimized parameters according to the Operator's Manual (version 3.5; Roche). Melting curves were checked for correctness and the size of the fragments was verified on agarose gels. In order to obtain a GAPDH standard curve, dilutions of GAPDH cDNA in a range from 3 × 10^-6 ^to 3 ng were subjected to LightCycler analysis. The absolute amount of GAPDH mRNA contained in each cartilage sample was obtained after LightCycler analysis by deduction from the GAPDH standard curve. A GAPDH standard was included in every LightCycler run, and the expression of each gene was normalized to the mRNA level of the housekeeping gene GAPDH in the corresponding sample (set as 100% GAPDH).

### Statistics

The mean and standard deviation of each variable were computed. The median and interquartile range were also calculated. A two-way analysis of variance was used to control for the side factor and for the time factor. The side factor was analyzed as the paired measure. Post-hoc tests were also performed to compare time points. Significant changes were depicted. Post-hoc test results were calculated (Scheffé tests) and a two-tailed *P *≤ 0.05 was considered significant. An explorative Mann–Whitney U-test and the Wilcoxon test were chosen to evaluate differences between two groups without alpha adjustment. Data analysis was performed with SPSS for Windows 11.0.1 (SPSS Inc., Chicago, IL, USA).

## Results

The age and body mass of the animals were similar in all experimental groups. All but one of the dogs, which was in the 24-week group, were male. One animal had to be excluded after euthanasia following severe injury, so that in the 12-week group only five animals were evaluated instead of six animals. There were no surgical complications, and none of the dogs showed clinical signs of OA, such as altered posture or motion. The animals did not show partially or totally restricted use of the ACLT-treated extremity. When opened, the joints were found to show no macroscopic signs of inflammation or cartilage degeneration at 6 and 12 weeks. In those joints opened 24 weeks after ATLC surgery incipient cartilage discoloration and softening were seen, which were slightly more frequent and pronounced at 48 weeks. Most dogs had an increased volume of synovial fluid in treated knees at the two later time points.

### Histological examination

**Figure 1:**
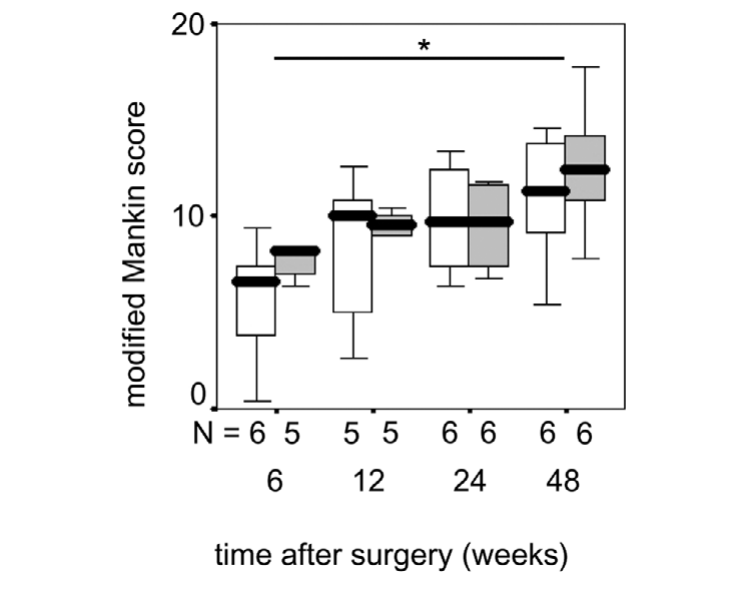
Box plot of histological grading in anterior cruciate ligament transection-treated knees at different times after surgery. Demonstrated are the median, standard deviation and interquartile range of the modified Mankin score data. White boxes, lateral tibial plateau; gray boxes, medial tibial plateau. * *P *< 0.05.

Histological features observed in cartilage from ACLT-treated knees and from control knees were similar to those described previously [3,4,18-20]. We found slight changes of the cartilage surface and chondrocytes at 6 weeks after surgery but such changes appeared also in part of the control samples. Decreased Safranin O staining and loss of zonal structure were noticeable in some specimens 12 weeks after ACLT, and were more pronounced in joints examined 48 weeks after surgery. Chondrocyte clustering and fissures never extending beyond the transitional zone were observed at 24 and 48 weeks, which never extended beyond the transitional zone. Variations in histopathological scores were evident within each group, indicating variations in the progression of osteoarthritic changes over time (Fig. 1). Median modified Mankin scores at 48 weeks (12.4) were significantly higher than those at 6 weeks (7.4) (*P *= 0.036), confirming progressive degeneration of cartilage in ACLT-treated knees. There was no obvious difference between the lateral and the medial compartments of the tibia or of the femur (48 weeks) and no progressive changes were obvious in the control knees over time. In summary, the ACLT-induced changes were progressive and consistent with early stages of OA.

### Gene expression analysis

The concentration of mRNA per sample was determined and used to calculate the absolute amount of mRNA per milligram of tissue wet weight. The mRNA content was similar in all study groups, providing no evidence for major differences in RNA extraction efficiency, tissue water content or overall transcriptional activity of cells between groups. The absolute amount of GAPDH mRNA per total mRNA was determined for each sample by LightCycler analysis using a GAPDH standard curve. A considerable variability of GAPDH per microgram of mRNA was noted that, according to values of the control side (right knee) and the ACLT side (left knee), primarily reflected differences between individuals. Absolute amounts of GAPDH levels per microgram of mRNA determined in all samples were compared by variance analysis comprising the factors time, side and treatment. Post-hoc tests were also performed to analyze differences between all groups. Statistical analysis revealed no significant differences in GAPDH levels between any of the study groups (controls, ACLT, locations, time).

Analysis of tenascinC and the chitinase-like molecules YKL39 and YKL40 was included in the present study because they have been related to human OA [21-23]. Since canine DNA sequence information on tenascinC, YKL39 and YKL40 was not available from public databases, degenerated primers were designed on the basis of multiple sequence alignment. Resulting PCR fragments for tenascinC (923 bp) and for YKL40 (665 bp) were sequenced, and they showed 90% and 83% nucleotide identity with the corresponding human cDNA sequence, respectively. The deduced protein sequence of the canine YKL40 fragment had 83% identical and 91% similar amino acids to human YKL40, and was only 48% identical to human YKL39. In spite of intense primer design and PCR analysis, it was not possible to obtain any fragments for YKL39 from dog cartilage. The dog genome database [24] also contained no sequence information with high homology to human YKL39. Interestingly, in spite of eightfold sequence coverage of the mouse genome, no YKL39 sequence is available from mouse databases and there is no orthologous gene at the locus corresponding to the one where human YKL39 is located. This suggested that mouse and dog lack the YKL39 gene, while YKL40 is present in the human, mouse, and dog.

### Early upregulation of col II, YKL40 and col I

**Figure 2:**
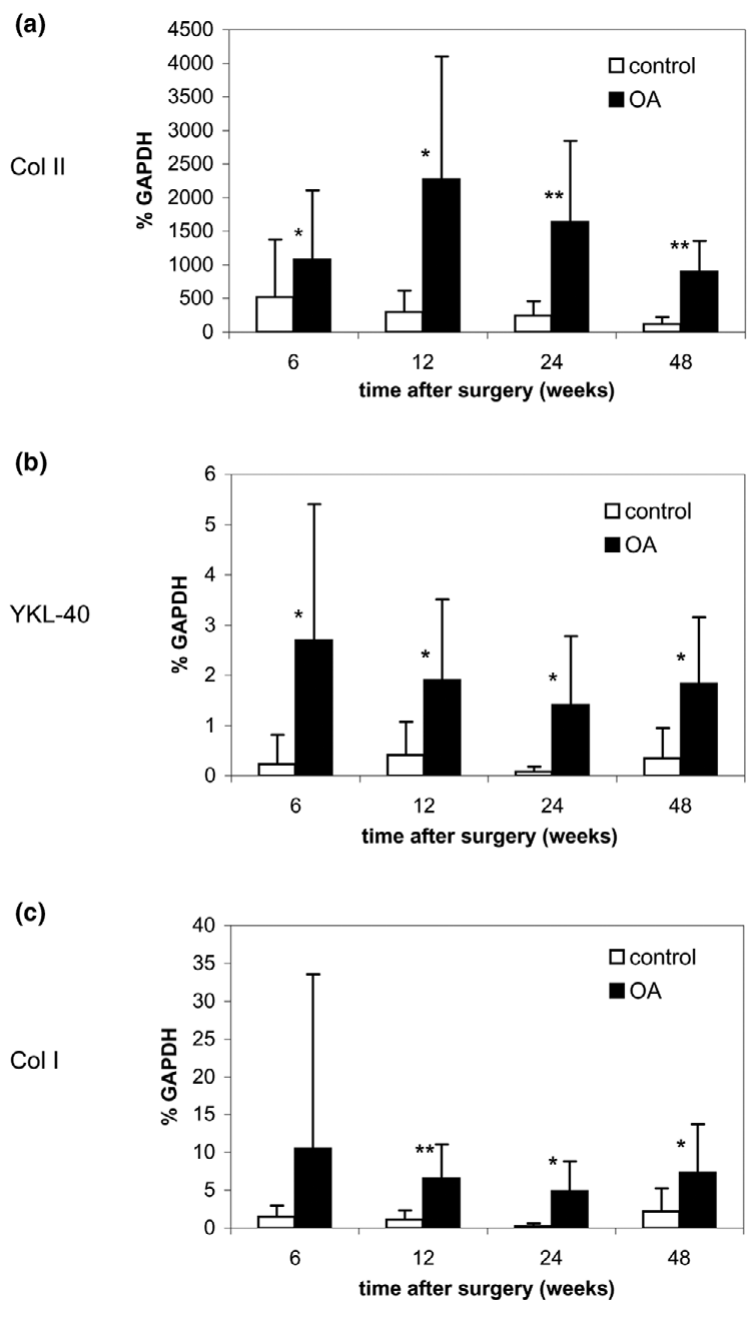
**(a) **Collagen type (col) II, **(b) **YKL-40 and **(c) **col I expression in cartilage of experimental osteoarthritis (OA) at different times after surgery. Shown are the mean relative expression levels of mRNA summarized for lateral and medial tibial plateau. * *P *< 0.05, ** *P *< 0.001.

Quantitative reverse transcriptase-PCR analysis of cartilage from the tibial plateau revealed significant upregulation of YKL40 and col II expression at all time points in ACLT-treated knees compared with the untreated joints. The median elevation was twofold to sevenfold for col II, and was threefold to 17-fold for YKL40 (Fig. 2a,2b). Gene expression of col I was significantly elevated at 12, 24 and 48 weeks after surgery in cartilage from the tibial plateau (Fig. 2c). Higher expression in OA samples was also evident at 6 weeks, but owing to a large standard deviation the difference did not reach statistical significance. There was no significant increase or decrease over time in the levels of col I or col II or of YKL40 in osteoarthritic cartilage. Femoral condyle samples were analyzed at 48 weeks after surgery. In keeping with our observations in the tibial plateau, expression of col II and of YKL40 was significantly higher in osteoarthritic cartilage than in control cartilage, while col I was highly variable (Table 1).

### Aggrecan, MMP13 and tenascinC expression

**Figure 3:**
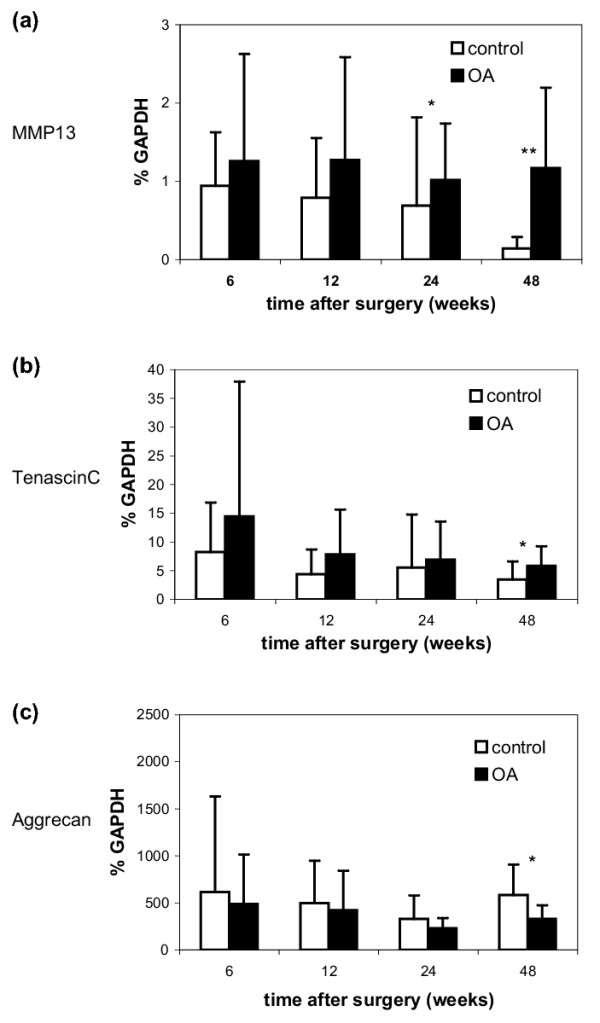
**(a) **MMP13, **(b) **tenascinC and **(c) **aggrecan expression in cartilage of experimental osteoarthritis (OA) at different times after surgery. Shown are the mean relative expression levels of mRNA summarized for lateral and medial tibial plateau. * *P *< 0.05, ** *P *< 0.001.

While mRNA levels for aggrecan tended to be lower in OA samples than in control samples, tenascinC and MMP13 levels tended to be higher (Fig. 3). Overall, for all time points, osteoarthritic and control knees did not differ significantly in aggrecan, MMP13 or tenascinC expression. At 24 and 48 weeks, however, MMP13 was significantly higher in ACLT-treated knees than in normal knees (Fig. 3a). Aggrecan mRNA levels showed relatively wide variation between samples taken from animals in the same study group, and median values tended to be slightly lower in osteoarthritic samples than in control samples. At 48 weeks after ACLT surgery the difference was twofold, and it reached statistical significance (*P *= 0.009) (Fig. 3c). In addition, elevated levels of tenascinC were seen in OA samples at 48 weeks after surgery (Fig. 3b). mRNA levels of aggrecan, tenascinC and MMP13 in the femur samples (48 weeks) were unchanged in OA samples compared with controls (Table 1).

### Region-specific differences in gene expression

**Figure 4:**
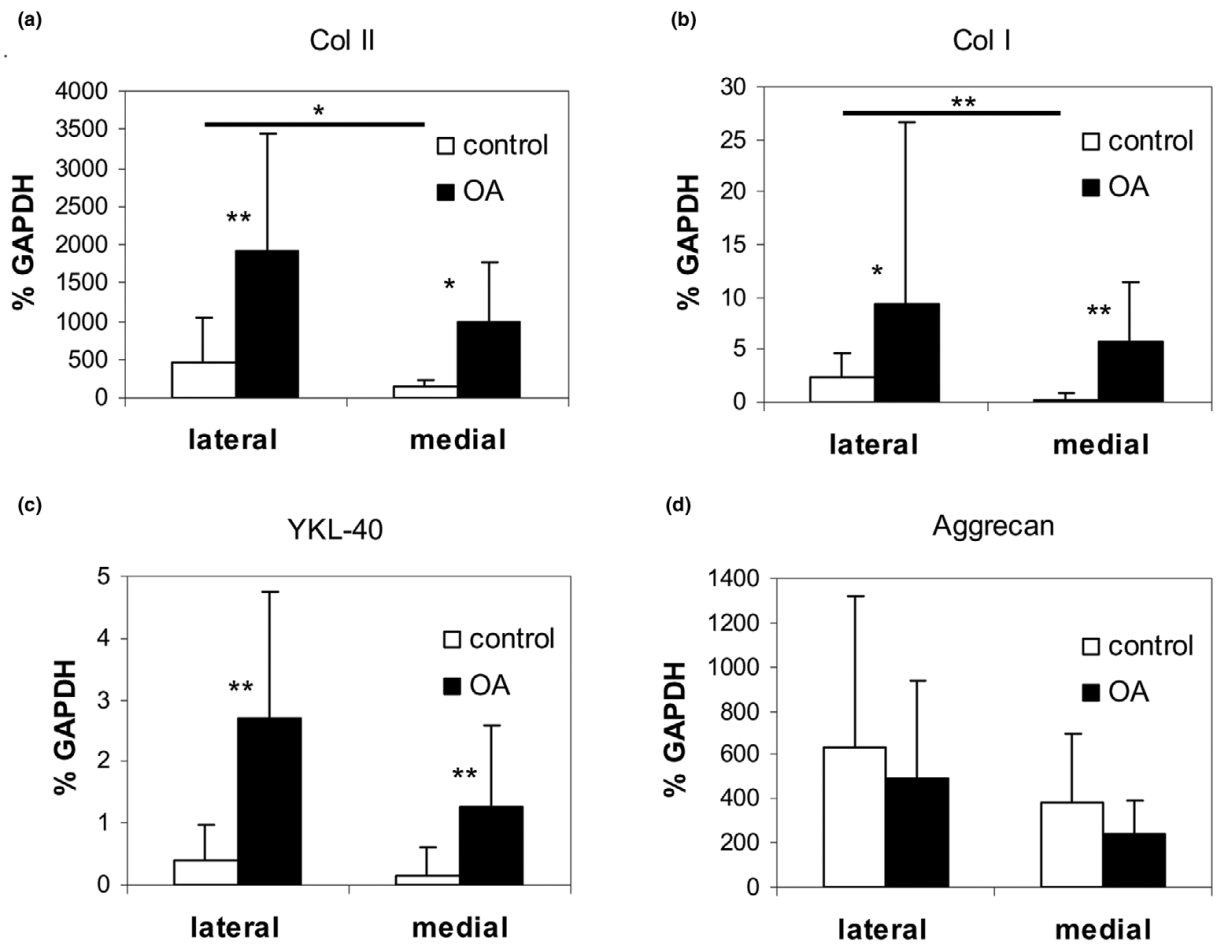
Local mRNA expression levels in cartilage of experimental osteoarthritis (OA): **(a) **collagen type (col) II, **(b) **col I, **(c) **YKL-40 and **(d) **aggrecan. Shown are mean relative expression levels of mRNA in lateral and medial tibial plateau summarized for 6, 12, 24 and 48 weeks. * *P *< 0.05, ** *P *< 0.001.

Significantly higher expression of col II, YKL40 and col I was evident in osteoarthritic samples than in normal cartilage samples from both the lateral and the medial tibial plateau (Fig. 4a,4b,4c) at all time points studied. The results for joints examined at all time points were pooled since no time effects were evident for col II, col I and YKL40. On average, the levels of col II in osteoarthritic joints were fourfold those in normal joints in the lateral compartment (*P *< 0.001) and were sevenfold those in normal joints in the medial compartment (*P *<0.001). YKL40 was elevated to 6.5-fold normal values (*P *< 0.001) in the lateral compartment and to eightfold in the medial compartment (*P *< 0.001). Expression of col I in the lateral compartment of ACLT-treated knees was fourfold that in control joints (*P *= 0.004); levels in the medial compartment were 22-fold normal (*P *< 0.001). The baseline expression of several genes tended to be higher in the lateral compartment than in the medial tibial compartment, reaching significance for col II expression (3.5-fold, *P *= 0.045) and for col I expression (ninefold, *P *< 0.001) (Fig. 4a,4c).

A similar tendency was seen for basic YKL40 and aggrecan expression, but not for MMP13 and tenascinC expression. In spite of region-specific baseline expression differences, robust generalized molecular effects were seen after ACLT surgery in canine knee cartilage, indicating a consistent regulation of the chondrocytes' response to an altered mechanical loading.

### No correlation between molecular changes and histological scoring

**Figure 5:**
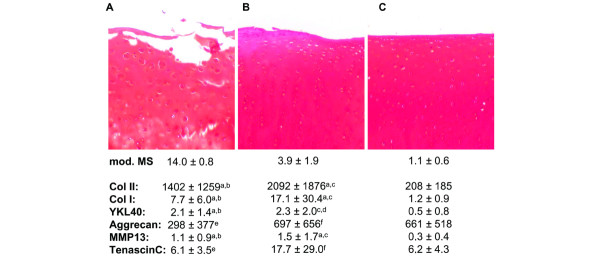
No correlation of molecular changes to the histological scoring. In order to directly correlate a certain histological outcome with gene expression independent of the study group or the location, samples adjacent to sections with **(a) **the highest (*n *= 8) or **(b) **the lowest (*n *= 7) modified Mankin score (mod. MS) in the anterior cruciate ligament transection-treated group were selected and were compared with **(c) **seven normal control samples with the lowest mod. MS in the study. Top, representative histological sample (magnification, 40 ×); bottom, corresponding mean and standard deviation of the mod. MS and of gene expression levels (% GAPDH expression). col., collagen type. ^a ^*P *< 0.05 versus (c), ^b ^no significant difference versus (b), ^c ^no significant difference versus (a), ^d ^*P *= 0.052 versus (c), ^e ^no significant difference versus (b) and (c), ^f ^no significant difference versus (a) and (c).

In order to correlate a particular histological outcome with gene expression independently of the study group or the location, samples adjacent to sections with extreme signs of chondrocyte cloning and with the highest (*n *= 8) or the lowest (*n *= 7) modified Mankin score in the ACLT-treated group were selected. The corresponding samples were compared with seven normal control samples (modified Mankin score 0–2). Comparison of extreme samples preselected for the highest histological scores (Fig. 5a) and the lowest histological scores (Fig. 5b) in the ACLT group will increase the statistical power to detect differences that would correlate with histological scoring. Nevertheless, we did not obtain molecular differences between these two ACLT groups, although both kept statistically significant molecular differences to the contralateral control group (Fig. 5c). Even samples with the lowest modified Mankin score in the ACLT group had already elevated col II (*P *= 0.004), col I (*P *= 0.007) and YKL-40 (*P *= 0.052) expression levels. Upregulation of col II, col I and YKL-40 was thus a very sensitive measure of cartilage degeneration that did not progress any further during the moderate advancement of cartilage degeneration examined in the present study.

## Discussion

Although OA has been well studied, many of the basic molecular mechanisms underlying its development are still unknown. The use of an animal model opens up the possibility of studying the early stages of disease progression and the regional pattern of matrix degradation by comparing diseased and healthy joints in the same individual. The results presented in this report demonstrate that ACLT leads to early and robust upregulation of the extracellular matrix molecule col II and of YKL40 in knee cartilage, which is independent of the time lapse since surgery, of the particular joint region studied and of the structural appearance of the extracellular matrix in adjacent histological sections. In addition, some evidence for a later change of gene expression of MMP13, aggrecan and tenascinC was obtained, which differed significantly from that on the control side by 24 and/or 48 weeks after surgery.

Our data might be interpreted as indicative of an early phase of OA development characterized by upregulation of col II, col I and YKL40, which is followed by a second phase of OA progression characterized by further upregulation of MMP13 and tenascinC. From a clinical point of view, the possibility of differentiating successive stages of OA progression by means of early and late disease markers is quite an attractive prospect. According to our data, col II and YKL40 are good candidates for use as robust, early and sensitive markers of joint instability. Although tenascinC and MMP13 may appear on a list of potential marker genes characterizing a second phase of OA, their expression will deserve further attention since no difference between ACLT samples with the lowest and the highest modified Mankin grades is yet obvious for these molecules. Such a distinction would be expected since cartilage degeneration progresses with time. Since the latest time point studied in our dog model is about 2–3 years before full-thickness loss of articular cartilage can be anticipated, the median and late alterations of gene expression cannot be expected to have occurred. Longer studies will be required to decide whether regulation of tenascinC and MMP13 indicate a further stage of molecular alterations in the Pond–Nuki model of OA development.

One of the major objectives of this project was to focus on local aspects of gene expression with reference to the histological grading of cartilage degeneration. Most strikingly, upregulation of col II, col I and YKL40 was pronounced in ACLT-treated knees even if the histological grading of adjacent tissue was only weak above control level and there was no progression with OA development (Fig. 5). Differing levels of severity or 'doses' of injury have been suggested by others as a possible reason for regional changes in mRNA expression [9]. Our study, however, lends little credence to this suggestion [10,13]. Knee instability is very likely to increase inappropriate mechanical loading in many parts of the joint and, in keeping with this, col II and YKL40 expression rose in all locations after ACLT in our study. Surprisingly, we detected significant differences in col II and col I expression between the lateral and the medial tibial plateau already in normal control knees, while levels of the housekeeping gene GAPDH did not differ significantly among the compartments at any time point of the study. Given that mechanical forces are modulators of chondrocyte metabolism, even physiologic loading differences may influence basal expression levels of sensitive genes and be the explanation for this effect. This is in line with reports of upregulation of col II and col I synthesis in chondrocytes in response to cyclic loading *in vitro *[25,26].

Elevated col II and aggrecan mRNA levels were reported previously in early-stage and median-stage canine OA after ACLT [9,10]. In contrast to our data, however, either a progressive [9] or a declining [10] elevation of col II RNA was observed over time in those studies. Beside the fact that semiquantitative methods have been used in these previous studies, the normalization of gene expression data will strongly influence the results [27]. While others decided to express changes in mRNA expression on a per cell basis by referring data to the DNA content of the tissue, we referred specific mRNA expression levels to expression of the housekeeping gene GAPDH. The strength of our method is that we can detect a specific regulation of cartilage-relevant molecules beyond generalized effects that may occur after ACLT, such as the overall activation of chondrocyte metabolism. Its weakness is that there is a risk that the housekeeping gene itself may be regulated by ACLT. Among a selection of housekeeping genes, GAPDH correlated best with total RNA content per milligram of tissue in dog cartilage [28]. Thorough statistical analysis of our data provided no evidence for either a regulation of total mRNA per milligram of tissue or a regulation of GAPDH in response to ACLT at any time point of the study. Given that the very weak trend to higher GAPDH levels after ACLT would become significant when data are referred on a per cell basis, the upregulation of col II, col I, YKL40, MMP13 and tenascinC per cell would be even more pronounced.

Elevated release of proteoglycan and col II protein degradation products into body fluids of ACLT-treated dogs [29] and of patients with advanced knee OA [30,31] indicate that loss of such molecules from the cartilage matrix makes a major contribution to the development of OA. Chondrocytes may sense this loss and respond to it with upregulation of col II mRNA levels, but they did not adapt mRNA levels for the aggrecan core protein accordingly in the present study. Although there is evidence for enhanced sulfate incorporation into proteoglycans of ACLT-treated cartilage versus normal dog cartilage [32-34], the identity of these molecules remained unknown. Enhanced mRNA levels for small proteoglycans like biglycan, decorin and fibromodulin after ACLT [11] may explain such observations, but due to the small sample size they unfortunately could not be included in the present study.

Human chondrocytes secrete two distinct chitinase-like molecules, called YKL39 and YKL40. While YKL40 has been linked with tissue remodeling, with joint injury and with *in situ *inflammatory macrophages [35-40], clinical correlates of YKL39 expression remained unknown. Enhanced expression of YKL39, but not of YKL40, was demonstrated in severe human OA cartilage [41,42]. However, in spite of reasonable effort, we have not been able to detect YKL39 in canine chondrocytes and databases. The upregulation both of YKL40 in early stages of dog OA and of YKL39 in late-stage human OA suggests, however, that chitinase-like molecules may have some function in cartilage remodeling and are potentially interesting marker molecules for osteoarthritic joint disease.

Human late-stage osteoarthritic cartilage from joint replacement surgery subjected to cDNA array analysis showed significantly elevated levels of col II, col I, chitinase precursor, tenascin and several matrix metalloproteinases, including MMP13, while aggrecan levels remain unaltered [43]. Taking into account the high donor-dependent variability in human samples, the lower sensitivity of the cDNA array technique than of quantitative PCR and the different time frames studied, the overlap between molecular alterations in natural human OA and experimental canine OA is considerable. This indicates that chondrocytes in dog cartilage respond to degenerative conditions by regulating the same genes as chondrocytes in human OA, and in a similar direction.

## Conclusion

In conclusion, upregulation of col II, col I and YKL40 was a very sensitive and robust response to the altered mechanical situation after ACLT surgery, which occurred quite independent of joint location and a certain grade of morphological cartilage degeneration. Levels did not progress any further during the moderate advancement of cartilage degeneration examined in the present study, and more progressed stages of cartilage degeneration may therefore rather be characterized by regulation of additional cartilage-relevant molecules like MMP13 and tenascinC.

We interpret the molecular alterations in natural human OA and experimental canine OA as considerable and we suggest that the Pond–Nuki model may be a suitable experimental model to unravel further basic anabolic and catabolic molecular mechanisms of relevance for human disease development.

## Abbreviations

ACLT = anterior cruciate ligament transection; bp = base pair; col = collagen type; GAPDH = glyceraldehydes-3-phosphate dehydrogenase; OA = osteoarthritis; PCR = polymerase chain reaction.

## Competing interests

The author(s) declare that they have no competing interests.

## Authors' contributions

HL participated in the design of the study, coordinated and assisted surgery, carried out the molecular analysis, evaluated histology and drafted the manuscript. WW participated in the design of the study, performed surgery and evaluated histological samples. MI assisted with surgery and evaluated histological samples. ES participated in the design and evaluation of molecular analysis, and contributed to the manuscript. WR conceived of the study, participated in its design, and contributed to histological and molecular data analysis and to the manuscript. All authors read and approved the manuscript.

## Figures and Tables

**Table 1:** mRNA expression levels of osteoarthritis-relevant genes in femoral condyles at 48 weeks

Group	Collagen type II	YKL-40	Collagen type I	Aggrecan	TenascinC	MMP13
Anterior cruciate ligament transection	1359.4* (1875.9 ± 1680.0)	1.0* (2.2 ± 2.6)	0.6 (1.4 ± 1.9)	472.0 (547.2 ± 448.6)	2.7 (11.9 ± 29.8)	0.2 (0.5 ± 0.5)
Control	121.3 (164.2 ± 150.2)	0.4 (0.3 ± 0.2)	0.7 (6.7 ± 13.2)	372.5 (434.3 ± 225.1)	1.0 (2.9 ± 2.8)	0.2 (0.7 ± 1.0)

Data presented as median (mean ± standard deviation).

* *P *< 0.05 versus control.

## References

[B1] Adams ME (1989). Cartilage hypertrophy following canine anterior cruciate ligament transection differs among different areas of the joint. J Rheumatol.

[B2] Pelletier JP, Martel-Pelletier J, Mehraban F, Malemud CJ (1992). Immunological analysis of proteoglycan structural changes in the early stage of experimental osteoarthritic canine cartilage lesions. J Orthop Res.

[B3] Pelletier JP, Martel-Pelletier J, Altman RD, Ghandur-Mnaymneh L, Howell DS, Woessner JF (1983). Collagenolytic activity and collagen matrix breakdown of the articular cartilage in the Pond–Nuki dog model of osteoarthritis. Arthritis Rheum.

[B4] McDevitt C, Gilbertson E, Muir H (1977). An experimental model of osteoarthritis; early morphological and biochemical changes. J Bone Joint Surg Br.

[B5] Little CB, Ghosh P, Bellenger CR (1996). Topographic variation in biglycan and decorin synthesis by articular cartilage in the early stages of osteoarthritis: an experimental study in sheep. J Orthop Res.

[B6] Vignon E, Bejui J, Mathieu P, Hartmann JD, Ville G, Evreux JC, Descotes J (1987). Histological cartilage changes in a rabbit model of osteoarthritis. J Rheumatol.

[B7] Pond MJ, Nuki G (1973). Experimentally-induced osteoarthritis in the dog. Ann Rheum Dis.

[B8] Sah RL, Yang AS, Chen AC, Hant JJ, Halili RB, Yoshioka M, Amiel D, Coutts RD (1997). Physical properties of rabbit articular cartilage after transection of the anterior cruciate ligament. J Orthop Res.

[B9] Matyas JR, Ehlers PF, Huang D, Adams ME (1999). The early molecular natural history of experimental osteoarthritis. I. Progressive discoordinate expression of aggrecan and type II procollagen messenger RNA in the articular cartilage of adult animals. Arthritis Rheum.

[B10] Matyas JR, Huang D, Chung M, Adams ME (2002). Regional quantification of cartilage type II collagen and aggrecan messenger RNA in joints with early experimental osteoarthritis. Arthritis Rheum.

[B11] Dourado GS, Adams ME, Matyas JR, Huang D (1996). Expression of biglycan, decorin and fibromodulin in the hypertrophic phase of experimental osteoarthritis. Osteoarthritis Cartilage.

[B12] Bluteau G, Conrozier T, Mathieu P, Vignon E, Herbage D, Mallein-Gerin F (2001). Matrix metalloproteinase-1, -3, -13 and aggrecanase-1 and -2 are differentially expressed in experimental osteoarthritis. Biochim Biophys Acta.

[B13] Bluteau G, Gouttenoire J, Conrozier T, Mathieu P, Vignon E, Richard M, Herbage D, Mallein-Gerin F (2002). Differential gene expression analysis in a rabbit model of osteoarthritis induced by anterior cruciate ligament (ACL) section. Biorheology.

[B14] Le Graverand MP, Eggerer J, Vignon E, Otterness IG, Barclay L, Hart DA (2002). Assessment of specific mRNA levels in cartilage regions in a lapine model of osteoarthritis. J Orthop Res.

[B15] Hellio Le Graverand MP, Reno C, Hart DA (2000). Heterogenous response of knee cartilage to pregnancy in the rabbit: assessment of specific mRNA levels. Osteoarthritis Cartilage.

[B16] Mankin HJ, Dorfman H, Lippiello L, Zarins A (1971). Biochemical and metabolic abnormalities in articular cartilage from osteo-arthritic human hips. II. Correlation of morphology with biochemical and metabolic data. J Bone Joint Surg Am.

[B17] Sakakibara Y, Miura T, Iwata H, Kikuchi T, Yamaguchi T, Yoshimi T, Itoh H (1994). Effect of high-molecular-weight sodium hyaluronate on immobilized rabbit knee. Clin Orthop.

[B18] Wenz W, Breusch SJ, Graf J, Stratmann U (2000). Ultrastructural findings after intraarticular application of hyaluronan in a canine model of arthropathy. J Orthop Res.

[B19] Caron JP, Fernandes JC, Martel-Pelletier J, Tardif G, Mineau F, Geng C, Pelletier JP (1996). Chondroprotective effect of intraarticular injections of interleukin-1 receptor antagonist in experimental osteoarthritis. Suppression of collagenase-1 expression. Arthritis Rheum.

[B20] Pelletier JP, Martel-Pelletier J, Ghandur-Mnaymneh L, Howell DS, Woessner JF (1985). Role of synovial membrane inflammation in cartilage matrix breakdown in the Pond–Nuki dog model of osteoarthritis. Arthritis Rheum.

[B21] Chevalier X, Groult N, Larget-Piet B, Zardi L, Hornebeck W (1994). Tenascin distribution in articular cartilage from normal subjects and from patients with osteoarthritis and rheumatoid arthritis. Arthritis Rheum.

[B22] Veje K, Hyllested-Winge JL, Ostergaard K (2003). Topographic and zonal distribution of tenascin in human articular cartilage from femoral heads: normal versus mild and severe osteoarthritis. Osteoarthritis Cartilage.

[B23] Pfander D, Rahmanzadeh R, Scheller EE (1999). Presence and distribution of collagen II, collagen I, fibronectin, and tenascin in rabbit normal and osteoarthritic cartilage. J Rheumatol.

[B24] Kirkness EF, Bafna V, Halpern AL, Levy S, Remington K, Rusch DB, Delcher AL, Pop M, Wang W (2003). The dog genome: survey sequencing and comparative analysis. Science.

[B25] Bonassar LJ, Grodzinsky AJ, Frank EH, Davila SG, Bhaktav NR, Trippel SB (2001). The effect of dynamic compression on the response of articular cartilage to insulin-like growth factor-I. J Orthop Res.

[B26] Sah RL, Kim YJ, Doong JY, Grodzinsky AJ, Plaas AH, Sandy JD (1989). Biosynthetic response of cartilage explants to dynamic compression. J Orthop Res.

[B27] Matyas JR, Adams ME, Huang D, Sandell LJ (1995). Discoordinate gene expression of aggrecan and type II collagen in experimental osteoarthritis. Arthritis Rheum.

[B28] Matyas JR, Huang D, Adams ME (1999). A comparison of various 'housekeeping' probes for northern analysis of normal and osteoarthritic articular cartilage RNA. Connect Tissue Res.

[B29] Matyas JR, Atley L, Ionescu M, Eyre DR, Poole AR (2004). Analysis of cartilage biomarkers in the early phases of canine experimental osteoarthritis. Arthritis Rheum.

[B30] Jung M, Christgau S, Lukoschek M, Henriksen D, Richter W (2004). Increased urinary concentration of collagen type II C-telopeptide fragments in patients with osteoarthritis. Pathobiology.

[B31] Garnero P, Conrozier T, Christgau S, Mathieu P, Delmas PD, Vignon E (2003). Urinary type II collagen C-telopeptide levels are increased in patients with rapidly destructive hip osteoarthritis. Ann Rheum Dis.

[B32] Carney SL, Billingham ME, Caterson B, Ratcliffe A, Bayliss MT, Hardingham TE, Muir H (1992). Changes in proteoglycan turnover in experimental canine osteoarthritic cartilage. Matrix.

[B33] Carney SL, Billingham ME, Muir H, Sandy JD (1984). Demonstration of increased proteoglycan turnover in cartilage explants from dogs with experimental osteoarthritis. J Orthop Res.

[B34] Sandy JD, Adams ME, Billingham ME, Plaas A, Muir H (1984). In vivo and in vitro stimulation of chondrocyte biosynthetic activity in early experimental osteoarthritis. Arthritis Rheum.

[B35] Johansen JS, Jensen HS, Price PA (1993). A new biochemical marker for joint injury. Analysis of YKL-40 in serum and synovial fluid. Br J Rheumatol.

[B36] Rejman JJ, Hurley WL (1988). Isolation and characterization of a novel 39 kilodalton whey protein from bovine mammary secretions collected during the nonlactating period. Biochem Biophys Res Commun.

[B37] Shackelton LM, Mann DM, Millis AJ (1995). Identification of a 38-kDa heparin-binding glycoprotein (gp38k) in differentiating vascular smooth muscle cells as a member of a group of proteins associated with tissue remodeling. J Biol Chem.

[B38] Krause SW, Rehli M, Kreutz M, Schwarzfischer L, Paulauskis JD, Andreesen R (1996). Differential screening identifies genetic markers of monocyte to macrophage maturation. J Leukoc Biol.

[B39] Boot RG, van Achterberg TA, van Aken BE, Renkema GH, Jacobs MJ, Aerts JM, de Vries CJ (1999). Strong induction of members of the chitinase family of proteins in atherosclerosis: chitotriosidase and human cartilage gp-39 expressed in lesion macrophages. Arterioscler Thromb Vasc Biol.

[B40] Renkema GH, Boot RG, Au FL, Donker-Koopman WE, Strijland A, Muijsers AO, Hrebicek M, Aerts JM (1998). Chitotriosidase, a chitinase, and the 39-kDa human cartilage glycoprotein, a chitin-binding lectin, are homologues of family 18 glycosyl hydrolases secreted by human macrophages. Eur J Biochem.

[B41] Steck E, Breit S, Breusch SJ, Axt M, Richter W (2002). Enhanced expression of the human chitinase 3-like 2 gene (YKL-39) but not chitinase 3-like 1 gene (YKL-40) in osteoarthritic cartilage. Biochem Biophys Res Commun.

[B42] Knorr T, Obermayr F, Bartnik E, Zien A, Aigner T (2003). YKL-39 (chitinase 3-like protein 2), but not YKL-40 (chitinase 3-like protein 1), is up regulated in osteoarthritic chondrocytes. Ann Rheum Dis.

[B43] Aigner T, Zien A, Gehrsitz A, Gebhard PM, McKenna L (2001). Anabolic and catabolic gene expression pattern analysis in normal versus osteoarthritic cartilage using complementary DNA-array technology. Arthritis Rheum.

